# Chemotherapy-induced polyneuropathy in cancer care—the patient perspective

**DOI:** 10.1007/s00520-023-07688-5

**Published:** 2023-03-27

**Authors:** K. Prager, K. Passig, O. Micke, B. Zomorodbakhsch, C. Keinki, J. Hübner

**Affiliations:** 1 Klinik Für Innere Medizin II, Universitätsklinikum Jena, Am Klinikum 1, 07747 Jena, Germany; 2 Klinikum Südstadt Rostock, Klinik Für Onkologie III, Südring 81, 18059 Rostock, Germany; 3 Department of Radio-Oncology, Franziskus Hospital, Kiskerstraße 26, 33615 Bielefeld, Germany; 4 üBAG/MVZ Onkologische, Kooperation Harz GbR, Kösliner Straße 14, 38642 Goslar, Germany

**Keywords:** Chemotherapy-induced polyneuropathy, CIPN, Peripheral neuropathy, Impact on daily life, Neuropathy pain, Quality of life

## Abstract

**Purpose:**

Chemotherapy-related polyneuropathy (CIPN) is a very common, often dose-limiting side effect that affects the patients’ quality of life. Treatment usually consists of a combination of medicinal, medical, and individualized treatment approaches, although the effectiveness of these therapies is insufficient for many patients. The aim of this article is to review and evaluate the impact of CIPN on patients’ daily lives and possible effective treatment approaches.

**Methods:**

A standardized questionnaire was developed based on ten anonymous telephone interviews with CIPN patients. The content of the questionnaire was divided into 5 categories: demographics, clinical presentation, everyday symptoms, treatment of CIPN symptoms, and medical care. Mostly closed questions were used but multiple choice and individual additions by free text answers were possible.

**Results:**

CIPN limits patients’ quality of life over a long period of time. In addition to diurnal and situational fluctuations, the emotional burden negatively affects patients’ daily lives in many ways. From the patients’ point of view, the individually implemented therapy measures were most effective in treating their complaints. But even the combination of different therapy methods insufficiently alleviates the symptoms of the patients.

**Conclusion:**

It is important and necessary to comprehensively inform patients about CIPN as a possible side effect, to point out prevention strategies, and to critically examine and evaluate different therapy approaches. In this way, misunderstandings of the doctor-patient relationship can be avoided. In addition, patient satisfaction and quality of life can be increased in the long term.

**Supplementary Information:**

The online version contains supplementary material available at 10.1007/s00520-023-07688-5.

## Introduction

Chemotherapy-induced polyneuropathies (CIPN), in addition to hematological toxicity, are a common adverse effects and occur acutely or chronically during chemotherapy [1, 2] while the exact incidence is not known [3, 4]. They often act as a therapy-limiting factor, necessitating the termination of ongoing chemotherapy. Depending on the duration of exposure and the cumulative dose, there are sensory-(motor) complaints, often associated with pain. After the end of chemotherapy, there is usually no further progression. Exceptions are platinum-containing chemotherapeutic agents, where progression—called coasting—is possible even after the end of therapy. The increasing use of chemotherapeutic agents in multiple combinations and interactions between them have hardly been studied. In addition, existing nerve damage or neuropathies—for example caused by diabetes mellitus—may worsen the severity of the neuropathy [1, 5].

The various chemotherapeutic agents induce specific patterns of symptoms [4]. Distal, sensorimotoric symptoms are typical for vinca alkaloids. Distal, sensitive symptoms are frequently occur for taxanes and purely sensitive symptoms for platinum derivatives [1, 6].

A reduction in the density of intradermal nerve fibers has been found in CIPN with accentuated sensory symptoms and pain. This reduction roughly correlates with the specified pain intensity [3]. As a result of the toxic effect of the drugs, damage primarily occurs to the large-caliber (myelinated) or mixed nerve fibers, while the small (non-myelinated) fibers are less affected [7].

In the case of damage to the small—little or unmyelinated—nerve fibers, the objective detection of electrophysiological changes is limited [3].

The chemotherapeutic agents hardly enter the central nervous system but are absorbed into the spinal ganglia and peripheral nerves and accumulate there. Various ion channels, receptors, and the mitochondria can be damaged and their expression altered over time. Activation of the innate immune system also appears to have an influence. Spontaneous discharges and an increased evoked response to natural stimuli are observed more frequently in the dorsal horn of the spinal cord [3]. However, electrophysiological abnormalities can be delayed or not occur at all [1, 5].

The diagnosis of CIPN is based on a clear anamnesis, appropriate anatomical distribution of symptoms and clinical or instrumental diagnostics. As a result, the symptoms can be classified “definitely” or “probably” as neuropathic [7].

The symptoms are characterized by losses of sensoric function, such as tingling paresthesia and they are often associated with pain. However, disturbances in vibration and touch sensitivity, temperature sensitivity, hyper- and allodynia, etc., can also be observed. In addition, motor symptoms such as impaired muscle reflexes [1], musculoskeletal pain [7], muscle weakness up to paralysis, and atrophy of individual muscle groups are possible [1].

Supportive care aims includes prevention, functional restoration, and symptomatic treatment of the complaints. As the individual course of CIPN is not foreseeable, the prominent question is whether to continue the treatment. Whereby the risk exists that the symptoms and deficits increase or even remain lifelong. However, both discontinuation of therapy and dose reduction may worsen the prognosis [1, 6]. A (complete) resolution of symptoms within weeks to years is possible [1].

Sensomotoric training was reported to reduce CIPN or prolong time to its appearance [8–10].

The focus of the standard treatment is a symptomatic—drug—therapy, which is similar to that of neuropathic pain. Other therapy methods, such as physiotherapy/exercise therapy, are important to restore functional limitations [1, 9, 11, 12].

In summary, CIPN has a very heterogeneous clinical picture. Its impact on patients is not only limited to the physical symptoms but affects everyday life [4, 6].

With our survey, we contribute more and refined data on the actual impact of CIPN in cancer patients.

In particular, the exact impact of symptoms on patients’ daily lives and their variability should be mapped more precisely. In conjunction with a comprehensive presentation of effective therapeutic approaches from the patient’s point of view (with a focus on individually feasible therapies), complementary possibilities of current therapeutic concepts are to be shown. For this purpose, it is equally important to break down the affected body parts in more detail. The aim is to enable a holistic treatment of the complaints. Another important concern for us was to provide a more detailed account of the doctor-patient relationship, which is essential not only for cancer treatment but also for the treatment of CIPN. Potential areas for improvement should be identified so that patients feel they are receiving the best possible care, further supporting their recovery.

## Methods and patients

### Questionnaire

To develop the questionnaire, a telephone interview about therapy-induced polyneuropathy was conducted with 10 representatives of the German cancer self-help group ILCO e.V. who were themselves affected.

The patients were interviewed by one person (KP) from November to December 2020 using standardized questionnaire (open questions). Through additional targeted follow-up questions—per preformulated keywords—individual focal points/key positions of the patients were identified and taken into account. With the patient’s consent, the interviews were temporarily and anonymously recorded—each record took time about 20-min and 90-min length.

In the interviews, the cancer disease, its treatment, the development and dynamics of the symptoms, and the effect on everyday life and family life were discussed. The informational discussion, the (medical) treatment, and satisfaction with it were also addressed.

The data from the telephone interviews were used to identify focal points of CIPN from the patients’ perspective. Among other things, response options describing symptoms or (drug) treatment could be added. The structure of the questionnaire was also directly influenced, for example, the doctor-patient relationship and the (doctor’s) handling of the disease were repeatedly addressed. In order to be able to depict these topics, we therefore decided to integrate a voluntary additional section into the questionnaire.

A standardized questionnaire was created from this content. The questionnaire consists of 5 sections:

1. Demographics: sex, age, duration of symptoms, cause of CIPN, cancer treated, diagnosis of polyneuropathy, and time elapsed to diagnosis.
2. Clinical appearance: initial symptoms, localization of abnormal sensations and pain, characterization of pain (6-point Likert scale “weak or hardly present (1)” to “unbearable (6)”) and symptoms (13 listed).
3. Everyday influence of the complaints: extent of limitations in (16 listed) everyday situations (3-point Likert scale “slightly (1)” to “strongly (3)”) and limitations in everyday life (4-point Likert scale “not at all (1)” to “always (4)”), daily variations (in 10 suggested situations) in symptom severity (3-point Likert scale “mildly increased (1)” to “much more severe (3)”).
4. Therapy of CIPN symptoms: efficacy of medical, medicinal and individual therapy methods using a 4-point Likert scale from “not (1)” to “strong (4).”
5. Medical care: information about CIPN and satisfaction with the information session (6-point Likert scale “completely (1)” to “not at all (6)”), time until the symptoms were addressed and medical reaction, satisfaction with the medical treatment (4-point Likert scale “not at all (1)” to “always (4)”), need for support.

Mostly closed questions were used, but individual additions via free text answers were also possible. The free text responses were used for 3 functions: as time data for age, duration of complaints, time to diagnosis. Average and minimum/maximum values were calculated from them. They were also used as additional options to the given answer choices and were grouped under the category “other.” Third, they were used for individual descriptions of initial symptoms, justifications of answer choices, or addressing issues of importance (to patients). Responses were shortened (if necessary) to key terms and counted. Often multiple selections were possible.

A distinction between acute and chronic PNP was not made. The exact time of the (cancer) treatment was also not described in detail. For documentation of localization, we used a figure of the body surface where the participants could mark localization of loss of sensibility or pain.

Patients who did not provide information for the respective question were not counted for the corresponding question. Therefore, different patient numbers resulted for the individual questions, which deviate from the total number of patients.

### Patients

The questionnaire was distributed to cancer patients in three cancer ambulatories and three rehabilitation hospitals as print version and online via the Haus der Krebs-Selbsthilfe, the umbrella organization of the leading national cancer self-help organization in Germany. Patients interested in participating could either fill in the developed questionnaire in SoSci Survey, a German online tool for conducting online surveys (https://www.soscisurvey.de/) or request a printed copy. The survey took place between April and November 2021.

Adult patients with existing PNP who completed the questionnaire analog or online and returned it by the deadline were included in the study. Patients who only looked at the questionnaire without giving an answer were excluded. If the questionnaire was sent after November 21, 2021, it was also not considered.

### Statistics

Our study is a mixed exploratory and sequential study with quantitative data collection. For the development of the questionnaire, qualitative data (based on telephone interviews with affected persons) were first collected and analyzed for orientation.

IBM SPSS Statistics Premium 28 and Excel Microsoft 365 were used for descriptive statistical analysis.

## Results

### Demographic data

The standardized and anonymous questionnaire was completed by 272 patients between April 17, 2021, and November 21, 2021. Of 240 responding patients, 48.8% (*N* = 117) were female and 51.3% (*N* = 123) were male. Age was between 37 and 88 years, the mean age was 65.3 years.

The type of cancer and the respective tumor treatment were reported by 146 patients. Almost two-thirds (*N* = 98; 67.1%) were treated for leukemia or lymphoma; 15.8% (*N* = 23) were treated for breast cancer.

All demographic data are shown in Table 1.

**Table 1:** Demographic data (*N* = 272)

	N (%) of valid answers*^2^
Age	*N* = 241
< 40	2 (0.8)
40–54	43 (17.8)
55–69	110 (45.6)
70–85	82 (34.0)
> 85	4 (1.7)
Gender	*N* = 240
Female	117 (48.8)
Male	123 (51.3)
Type of cancer*	*N* = 146
Leukemia/lymphoma	98 (62.8)
Breast cancer	23 (14.7)
Prostate cancer	9 (5.8)
Colon cancer	8 (5.1)
Pancreatic cancer	5 (3.2)
Lung cancer	3 (1.9)
Ovarian cancer	3 (1.9)
Others*^3^	7 (4.5)

*Ten patients reported more than one cancer

*^2^Deviations from 100% due to rounding

*^3^2 × Thyroid cancer; 1 × each uterine, bladder, peritoneal, esophageal, and oropharyngeal carcinoma

### Basic data on CIPN

The symptoms of CIPN occurred for an average of 73.3 months with a range from 2 weeks to 27 years (see Table 2).

**Table 2:** Clinical appearance of CIPN

	N (% of valid answers)*
Duration of the complaints (*N* = 165)	
0–3 months	4 (2.4)
4–6 months	7 (4.2)
7–12 months (max. 1 year)	15 (9.1)
13–36 months (> 1 y, max. 3 y)	39 (23.6)
37–60 months (> 3 y, max. 5 y)	31 (18.8)
61–120 months (> 5 y, max. 10 y)	45 (27.3)
121–180 months (> 10 y, max. 15 y)	12 (7.3)
181–240 months (> 15 y, max. 20 y)	7 (4.2)
> 240 months (> 20 years)	5 (3.0)
Cause (*N* = 176)	
Cancer treatment	124 (70.5)
Diabetes	5 (2.8)
TNF inhibitors	1 (0.6)
Others	18 (10.2)
Unclear	28 (15.9)
Time to diagnosis (*N* = 130)	
0–2 weeks	48 (36.9)
3–5 weeks	14 (10.8)
6–10 weeks	18 (13.9)
11–20 weeks	22 (16.9)
21–30 weeks	9 (6.9)
31–40 weeks	7 (5.4)
41–50 weeks	2 (1.5)
51–100 weeks	6 (4.6)
> 100 weeks	4 (3.1)
Diagnosis (*N* = 147)	
Oncologist	62 (42.2)
Neurologist	53 (36.1)
General practitioner	13 (8.8)
Diabetologist	5 (3.4)
Others	14 (9.5)

*Deviations from 100% due to rounding

More than two-thirds (70.5%) of the 176 patients named cancer drugs as the cause, while the exact origin of CIPN is unclear in 15.9% (*N* = 28). Other comorbidities which may induce or aggravate polyneuropathy were named by 24 (13.6%) patients.

The diagnosis was usually made by an oncologist or neurologist, more rarely by general practitioners or other specialists, such as diabetologists, orthopedists, or pain therapists. Eight patients reported that they did not have yet received an official diagnosis or even they diagnosed the polyneuropathy themselves. Time to diagnosis describes the length of time from the onset of polyneuropathic symptoms and the diagnosis of polyneuropathy. Accordingly, time to diagnosis was considered independently of the cancer disease. It took an average of 15 weeks (about 3.5 months) from the onset of symptoms to diagnosis; the longest period was 120 weeks (over 2 years). The median was 6 weeks, almost two-thirds was diagnosed within the first 4 months.

#### Localization

In the 185 responding patients, the sensory disturbances were mostly symmetrical and more frequently localized on the front of the body than on the back (see Table 3). In the case of complaints of feet area, these often radiate into the lower legs, as is typical for stockings. The thighs were occasionally also affected (up to 5%); complete unilateral or bilateral leg involvement was denied. One-third of the patients named sensory disturbances in the area of the upper extremities. These were also mostly symmetrical and often localized in the area of the acras and the front of the body or the inner surface of hands and arms. Symptoms in the lower and upper arms played only a minor role. If complaints were localized on the back of the finger or hand, they usually also appeared on the corresponding inner side.

**Table 3:** Localization of the symptoms (*N* = 185)*

		Sensory disturbances (*N* = 185)			Pain (*N* = 79/185)		
		ventral	dorsal	Ventral + dorsal	ventral	dorsal	Ventral + dorsal
Toes	L (left)	75 (40.5%)	49 (26.5%)	35 (18.9%)	30 (16.2%)	33 (17.8%)	19 (10.3%)
	R (right)	68 (36.8%)	51 (27.6%)	32 (17.3%)	32 (17.3%)	28 (15.1%)	18 (9.7%)
Toes on both sides				30 (16.2%)			16 (8.7%)
Foot	L	42 (22.7%)	47 (25.4%)	24 (13.0%)	19 (10.3%)	22 (11.9%)	11 (6.0%)
	R	49 (26.5%)	51 (27.6%)	27 (14.6%)	25 (13.5%)	30 (16.2%)	17 (9.2%)
Foot on both sides				17 (9.2%)			10 (5.4%)
Toes + foot on both sides				9 (4.9%)			4 (2.2%)
Lower leg	L	42 (22.7%)	35 (18.9%)		12 (6.5%)	19 (10.3%)	
	R	39 (21.1%)	33 (17.8%)		13 (7.0%)	21 (11.4%)	
Upper thigh	L	7 (3.8%)	3 (1.6%)		10 (5.4%)	4 (2.2%)	
	R	9 (4.9%)	6 (3.2%)		8 (4.3%)	4 (2.2%)	
Finger	L	57 (30.8%)	36 (19.5%)	30 (16.2%)	13 (7.0%)	10 (5.4%)	8 (4.3%)
	R	61 (33.0%)	36 (19.5%)	30 (16.2%)	13 (7.0%)	9 (4.9%)	8 (4.3%)
Finger on both sides				27 (14.6%)			7 (3.8%)
Palm	L	26 (14.1%)	11 (6.0%)	11 (6.0%)	12 (6.5%)	6 (3.2%)	4 (2.2%)
	R	27 (14.6%)	14 (7.6%)	14 (7.6%)	12 (6.5%)	7 (3.8%)	5 (2.7%)
Palm on both sides				11 (6.0%)			4 (2.2%)
Finger + palm on both sides				7 (3.8%)			2 (1.1%)
Forearm	L	2 (1.1%)	2 (1.1%)		6 (3.2%)	4 (2.2%)	
	R	1 (0.5%)	3 (1.6%)		5 (2.7%)	4 (2.2%)	
Upper arm	L	1 (0.5%)	1 (0.5%)		4 (2.2%)	3 (1.6%)	
	R	1 (0.5%)	3 (1.6%)		4 (2.2%)	3 (1.6%)	

*Deviations from 100% due to rounding

Painful areas were marked in 79 of 185 patients (42.7%), mostly symmetrical and more often localized in the lower extremities. On the front of the lower extremities, the toes were most frequently affected, while pain in the ascending regions is reported less and less. The back of the body was just as often affected or slightly more frequently in the area of the soles of the feet and calves. One or both legs never hurt completely, a bilateral spread from the toes to the lower legs was once described.

Upper extremity pain is rarely described, most commonly in the fingers and hands and more commonly in the front of the body. Spread to one or both complete arms were denied; only 1 patient had bilateral complaints from the fingertips to the forearm. Two patients reported pain in the entire hands and feet.

### Symptoms as described by the patients

The 164 patients who gave a free text answer about the first manifested symptoms of their CIPN used an average of 2.2 descriptions with a maximum of 9 entries. The first symptoms were mainly localized in the feet (in 50%) and/or hands (24%). The arms (*N* = 7; 4.2%) were mostly spared, while the symptoms of the feet more often spread upwards (*N* = 47; 28.7%).

**Fig. 1:**
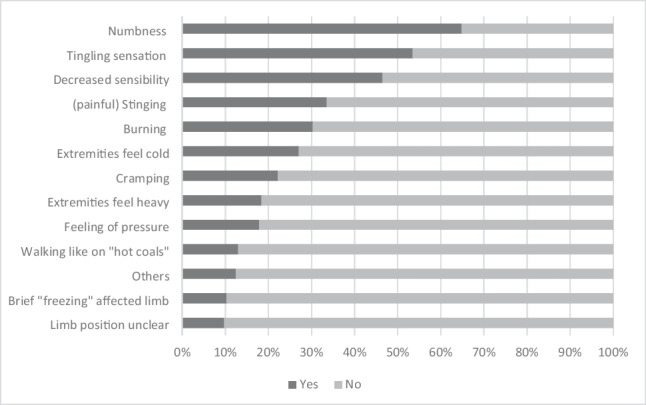
Symptoms as described by the patients (*N* = 185)

The patients marked an average of 4 and a maximum of 11 different descriptions for their symptoms, 41 of 185 patients did not agree with any description. Most frequently (*N* = 66; 35.7%), 4–6 symptoms were described. Numbness (*N* = 120), a tingling sensation (*N* = 99), or a decreased sensibility (*N* = 86) in the affected body regions was mentioned most frequently by far (see Fig. 1).

About two-thirds of the 120 patients (*N* = 85; 70.8%) who reported numbness also reported tingling. The symptom combination “numbness” and “decreased sensibility” was presented by 77 patients (64.2%). Tingling, numbness, and decreased sensibility occurred at the same time in 56 patients (46.7%). About 15% described, in addition to these three (most common) complaints, a (painful) stinging (*N* = 28), or burning (*N* = 27) sensation as a symptom.

The concomitant occurrence of the symptom “pain” was described by 121 patients. About ^1^/_3_ overall described them as “weak” (*N* = 11; 9.1%) or “mild” (*N* = 28; 23.1%). About ^1^/_3_ (*N* = 42; 34.7%) described their pain as “moderately severe.” The rest called them “strong” (*N* = 21; 17.4%), “very strong” (*N* = 12; 9.9%) or “unbearable” (*N* = 7; 5.8%).

### Impact of CIPN on daily life

The patients often felt restricted in everyday life due to their symptoms; only five patients (3.7%) denied this. About a quarter (27.0%; *N* = 37) experienced limitations “occasionally” due to the symptoms, while 62 of the 137 patients (45.3%) often experienced limitations. Being negatively affected by polyneuropathy at any time was affirmed by 33 patients (24.1%).

**Fig. 2:**
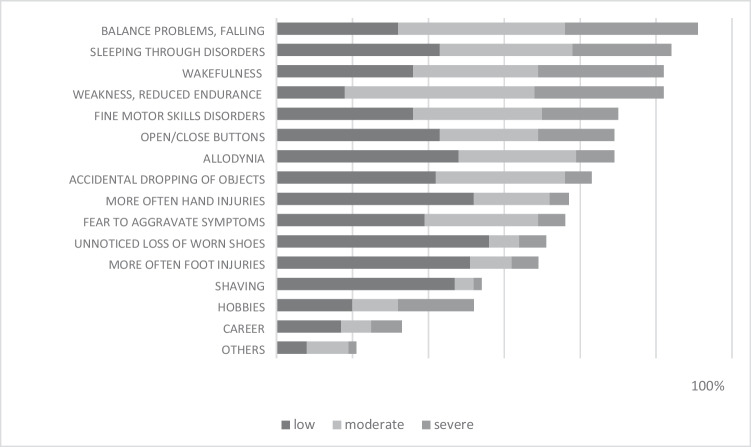
Impact of CIPN on daily life (*N* = 145)

Complaints in daily life were mentioned by 135 patients (see Fig. 2). An average of 9 out of 16 situations in which they felt impaired by their disease were selected from the list. Daily life was heavily or moderately influenced by increased stumbling/falling or general balance problems (*N* = 111; 82.2%). These was followed by increased weakness/reduced endurance (*N* = 102; 75.6%). In this context, 76 patients (56.3%) reported that they are afraid of further aggravating their symptoms through overexertion. Wakefulness or sleeping through disorders were reported by 113 patients (83.7%); 93 (68.9%) indicated both forms. The impact on sleep quality was mixed and rated slightly more important in case of wakefulness.

Up to two-thirds reported motor skills disorders (*N* = 90; 62.1%), repeated injuries to the hands (*N* = 77; 53.1%), dropping objects (*N* = 83; 57.2%), or an increased sensation of pain (*N* = 89; 61.4%). Yet the relevance to their everyday life was usually low.

The need for more support in coping with the disease and its consequences was expressed by 103 of 123 patients (82.1%). The points of criticism were widely spread. In addition to earlier recognition or diagnosis, from the patient’s point of view (interdisciplinary), treatment had to be significantly more effective and medical care should improve. Thus, among other things, “aids” to compensate for, e.g., tactile/grasp insecurities (“‘aids’ to compensate for, e.g., touch/grasp insecurities”) or more recommendations for better treatment (“treatment recommendations with a neurologist”) are required. The demand for better or more comprehensive clarification and increased research was expressed again and again.

### Factors aggravating the symptoms of CIPN

**Fig. 3:**
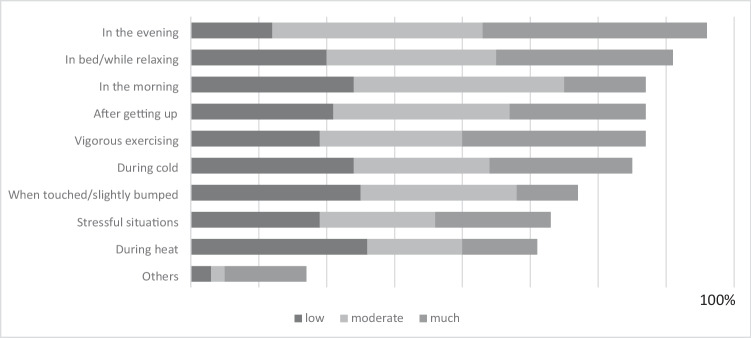
Factors aggravating the symptoms of CIPN (*N* = 98)

The majority of the 147 patients (*N* = 103; 70,07%) reported that their symptoms fluctuated depending on the situation and/or time of day and, as a result, showed variable symptom severity. Of the 147 patients, 98 named specific situations with aggravated symptoms, most frequently between 9 and 10 (*N* = 36; 36.7%) different situations (see Fig. 3). The symptoms were often worse “in the evening” (*N* = 76; 77.6%) and “while relaxing” (*N* = 71; 72.5%). A mostly moderate or pronounced negative influence on their symptoms by vigorous physical activity, after getting up or in the morning was described by 67 patients (68.4%). The 65 patients (66.3%) who described being influenced by cold estimated the negative effects differently.

The medical treatment and its success were evaluated by 120 patients (see Table 4). They received an average of 3 and a maximum of 7 (*N* = 13; 10.8%) different treatments. With 71 mentions (59.2%), treatment with gymnastics was reported most frequently, which was mostly found to be somewhat (*N* = 29; 40.9%) or very helpful (*N* = 17; 23.9%). Physiotherapy was used with comparable frequency (*N* = 67; 55.8%) and effectiveness. A rehabilitation program was attended by 58 patients (48.3%), most of whom rated it as somewhat (*N* = 24; 41.4%) or very (*N* = 16; 27.6%) symptom relieving. Electrotherapy was performed in 48 patients, the majority rated this as not (*N* = 19; 39.6%) or slightly (*N* = 10; 20.8%) positive. Psychological care or lymphatic drainage was given to 36 patients, respectively, while the lymphatic drainage was classified somewhat more positively. Satisfaction with a completed cure was mixed among the 33 patients (27.5%).

**Table 4:** Treatments and their efficacy as reported by the patients

	Not helpful	Hardly helpful	Somewhat helpful	Very helpful	Total mentions
Efficacy medical therapy (*N* = 120)					
Gymnastics	8 (11.3%)	17 (23.9%)	29 (40.9%)	17 (23.9%)	71 (59.2%)
Physical therapy	9 (13.4%)	15 (22.4%)	28 (41.8%)	15 (22.4%)	67 (55.8%)
Rehab	9 (15.5%)	9 (15.5%)	24 (41.4%)	16 (27.6%)	58 (48.3%)
Electricity therapy	19 (39.6%)	10 (20.8%)	12 (25.0%)	7 (14.6%)	48 (40.0%)
Lymphatic drainage	10 (27.8%)	4 (11.1%)	15 (41.7%)	7 (19.4%)	36 (30.0%)
Psychological care	12 (33.3%)	10 (27.8%)	12 (33.3%)	2 (5.6%)	36 (30.0%)
Cure	10 (30.3%)	4 (12.1%)	11 (33.3%)	8 (24.2%)	33 (27.5%)
Others	4 (16.0%)	0 (0.0%)	4 (16.0%)	17 (68.0%)	25 (20.8%)
Efficacy (oral) drug therapy (*N* = 91)					
Vitamin B12	8 (21.1%)	16 (42.1%)	12 (31.6%)	2 (5.3%)	38 (41.8%)
Painkillers	12 (34.3%)	7 (20.0%)	14 (40.0%)	2 (5.7%)	35 (38.5%)
Pregabalin	6 (23.1%)	4 (15.4%)	10 (38.5%)	6 (23.1%)	26 (28.6%)
Others	3 (17.7%)	4 (23.5%)	6 (35.3%)	4 (25.5%)	17 (19.8%)
Antidepressants, psychotropics	4 (26.7%)	3 (20.0%)	8 (53.3%)	0 (0.0%)	15 (16.5%)
Alpha-lipoic acid	3 (60.0%)	2 (40.0%)	0 (0.0%)	0 (0.0%)	5 (5.5%)
Efficacy individual therapy (*N* = 131)					
Sports, physical exercises	10 (9.4%)	21 (19.8%)	41 (38.7%)	34 (32.1%)	106 (80.9%)
Distractions (hobbies, work, meeting with family/friends, watching movies, etc.)	7 (7.5%)	18 (19.4%)	46 (49.5%)	22 (23.7%)	93 (71.0%)
Mobility and balance exercises	8 (9.0%)	23 (25.8%)	37 (41.6%)	21 (23.6%)	89 (67.9%)
Elevating of the feet	21 (23.6%)	21 (23.6%)	35 (39.3%)	12 (13.5%)	89 (67.9%)
Talking about complaints	28 (32.9%)	27 (31.8%)	20 (23.5%)	10 (11.8%)	85 (64.9%)
Strengthening exercises	12 (14.3%)	20 (23.8%)	32 (38.1%)	20 (23.8%)	84 (64.1%)
Heat	22 (27.5%)	14 (17.5%)	24 (30.0%)	20 (25.0%)	80 (61.1%)
Cold	43 (60.6%)	8 (11.3%)	16 (22.5%)	4 (5.6%)	71 (54.2%)
Relaxation exercises, mental training	8 (12.7%)	15 (23.8%)	23 (36.5%)	17 (27.0%)	63 (48.1%)
Eye control (during movements)	12 (17.4%)	11 (15.9%)	18 (26.1%)	20 (29.0%)	61 (46.6%)
Others	1 (11.1%)	0 (0.0%)	3 (33.3%)	5 (55.6%)	9 (6.9%)
Satisfaction with current treatment (*N* = 78)					
	Not at all	Seldom	Often	Always	
Oncologist	14 (26.9%)	13 (25.0%)	17 (32.7%)	8 (15.4%)	52 (66.7%)
General practitioner	13 (33.3%)	15 (38.5%)	8 (20.5%)	3 (7.7%)	39 (50.0%)
Neurologist	12 (26.7%)	17 (37.8%)	10 (22.2%)	6 (13.3%)	45 (57.7%)
Diabetologist	7 (63.6%)	2 (18.2%)	2 (18.2%)	0 (0.0%)	11 (14.1%)
Others	4 (30.8%)	2 (15.4%)	6 (46.1%)	1 (7.7%)	13 (16.7%)

*Deviations from 100% due to rounding

Current medication treatment of their CIPN was confirmed by 91 patients. Vitamin B12 is used most frequently (*N* = 38; 41.8%)—primarily as required—usually with a slight improvement (*N* = 16; 42.1%) of the symptoms. Often (*N* = 35; 38.5%) the complaints were treated with painkillers—usually taken when needed. The effect was assessed differently, only 2 patients (5.5%) attested a strong effectiveness. Somewhat less frequently (*N* = 26; 28.6%) pregabalin was taken (usually daily), about 3/5 noted moderate to very good effectiveness. The daily intake of antidepressants/psychotropics (*N* = 15; 16.5%) usually brought about a moderate improvement, which was not confirmed with alpha-lipoic acid (*N* = 5; 5.5%).

In order to alleviate the symptoms of their polyneuropathy in the best possible way, patients also used individually performed exercises. Up to 11 different, on average 6, treatment approaches were attempted by 131 patients. Most frequently (*N* = 55; 42.0%), 8 to 10 measures to relieve symptoms were tested. About 80% of the patients (*N* = 106; 80.9%) used sports and physical exercises, the assessment was mostly positive. Slightly less frequently (*N* = 93) distractions are said to reduce the symptom burden, which is said to be more effective than sport. Mobility and balance exercises or an elevating of the feet were performed by 89 patients (67.9%), as the former were rated more effective.

Almost two-thirds tried to achieve relief through strengthening exercises (*N* = 84; 64.1%) or by exchanging information with other affected persons and the family (*N* = 85; 64.9%). About 60% of the patients attested that strengthening exercises had a slight (*N* = 20) or pronounced (*N* = 32) positive effect, almost ¼ (*N* = 20; 23.8%) had a slightly positive effect. The patients rated the exchange with other affected patients or the conversation with the family as hardly positive.

About 3/5 of the patients (*N* = 80; 61.01%) tried to alleviate their symptoms with heat, about half (*N* = 71; 54.2) with cold. While warmth mostly proved to be (slightly) positive, cold was mostly not perceived as alleviating. Almost half (*N* = 63; 48.1%) used relaxation exercises and mental training, around two-thirds drew a rather positive conclusion. Less frequently (*N* = 61; 46.6%), increased eye control during movements to avoid falls or similar accidents was mentioned. The impact on daily life was described as very or rather relevant by two-thirds. Other therapeutic approaches listed were massage treatments and a consciously controlled execution of movements.

Current treatment of their CIPN symptoms by a physician was described by 78 patients. Most patients reported one physician in charge (*N* = 34; 43.6%), most frequently the oncologist. The satisfaction with the treatment was heterogeneous. Described by 39 patients (50.0%) treatment by a general practitioner was mostly (*N* = 15) or always (*N* = 13) dissatisfied.

### Communication with the physician

#### Pre-chemotherapy consultation

**Fig. 4:**
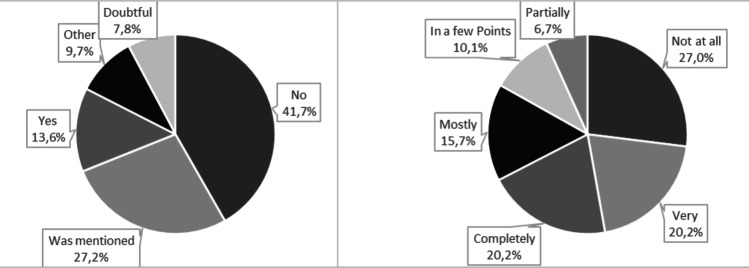
Explanation of CIPN (*N* = 103) + satisfaction with the educational interview before chemotherapy (*N* = 89)

The question whether CIPN was named as a side effect and explained in an understandable way during the consultation before chemotherapy was answered by 103 patients (see Fig. 4). Only 14 patients (13.6%) answered this question in the affirmative; in another 28 (27.2%), the term was mentioned in the educational interview. Most frequently, the CIPN was not explained (*N* = 43; 41.7%).

Satisfaction with the explanations rated 89 patients, 36 (40.5%) of them were completely or very satisfied. The explanations in these cases were rated as insightful, detailed, and easy to understand. Fourteen patients (15.7%) were mostly satisfied. Negative mentioned was the incomplete naming of possible side effects (e.g., the CIPN), positive was the good comprehensibility. Because the treatment in principle was explained but side effects and their consequences not in detail, 6 patients (6.7%) were partially satisfied. Nine patients (10.1%) were satisfied in a few points; the explanation was very short and incomplete due to excessive demands and lack of time by the physicians. Because of the insensitive and incomplete explanations, 24 patients (27.0%) were not satisfied at all.

#### Communication with the physician after start of CIPN

When and why they first discussed their polyneuropathic complaints with their treating physicians was stated by 86 patients. More than half (*N* = 48; 55.8%) did it immediately after the onset of symptoms. Twelve patients reported their symptoms after 1–3 months due to a delayed perception or lack of improvement, others after 6 months or even 1 year. The urging of family and friends also prompted some patients to address their complaints. Ten patients (11.6%) did not name their complaints because they had not primarily perceived them as a side effect. The remaining 13 patients (15.1%) expressed their polyneuropathy symptoms for the first time in a different way.

The reactions of the respective doctors to their complaints were described very differently by 88 patients. In around one-third (*N* = 28; 31.8%), the chemotherapy was adjusted or ended; in case of 18 patients (20.5%), the symptoms were treated and the cause sought. Some (*N* = 9; 10.2%) were only helped after an explicit request, while 21 patients (23.9%) were ignored and the current chemotherapy continued unchanged. In case of 8 patients (9.1%), the direct request for help was ignored or not responded. In the free text response, 23 patients (26.1%) described further reactions from the medical staff, ranging from understanding to rejection.

## Discussion

CIPN is a disease that affects and limits patients’ quality of life in a number of ways and over a long period of time. The sensory disturbances were mostly symmetrical and occurred most frequently in the area of the feet—as typical for stockings—and less frequently in the area of the hands. The distribution pattern was similar for the occurrence of pain.

The most commonly described symptom was numbness (in about two-thirds of patients). Tingling or decreased sensation occurred, often simultaneously, in about half of the patients. Many patients also reported pain, with about one-third describing each as moderate or at least severe.

Almost all patients reported several everyday situations that were mostly little or moderately negatively affected by the complaints. More than three-quarters reported problems with sleeping, walking, balance, or strength endurance. Many patients reported diurnal variations, especially an increase of symptoms toward evening or in relaxed situations.

For various reasons, almost all patients feel inadequately supported in the treatment of their CIPN and its consequences. Of the medical therapies, gymnastics and physiotherapy were rated most positively. The effect of medication is predominantly described as disappointing. As individual therapy measures, sports and stretching exercises have the highest value and are used regularly by about ¾ of the patients. However, balance exercises and communication with family and friends about the complaints also play an important role.

In most cases, the complaints were promptly addressed with the treating physicians, whereupon the chemotherapy was adjusted or terminated in about one-third. In about one-third of the patients, medical treatment was delayed or not given at all. Treatment is mostly provided by the oncologist, neurologist, and/or general practitioner, but patients are often not satisfied with the quality.

Information about CIPN as a possible side effect was often not given or not provided sufficiently; the overall satisfaction with the information was divided.

Patients with CIPN are affected by a variety of symptoms of different qualities that occur simultaneously, for example, from paresthesia to numbness. These complaints in turn affect everyday life and limit quality of life. Even if a single symptom only has a minor effect, this affects many different everyday situations and always remembers the patient on his/her disease. This may lead to a pronounced emotional burden, which may also impair quality of life as a whole [13].

Furthermore, as CIPN is not visible and hardly measurable, many patients struggle with a lack of acceptance or acknowledgment of their complaints and limitations in (work and) everyday life.

The most commonly used PROMs (patient-reported outcome measures) to identify CIPN are the QLQ-CIPN20 and the FACT/GOG-Ntx questionnaire [14, 15]. Among other things, they ask about the symptoms of the last 7 days, while, in our case, in addition to the current complaints, the onset of symptoms (and their presentation) was asked separately. In contrast to the two already established PROMs, our questionnaire asked about the localization of the complaints not only in the hands and feet, but in the entire body. We also asked in more detail about the respective impact on the daily lives of those affected [14].

Our questionnaire attempts to serve as a link between the pure symptom presentation and the psychological/everyday burden. Like other existing questionnaires, it cannot capture all facets of the disease and its consequences. A possible application would therefore be an initial rough query of the symptoms, their consequences in everyday life, and the identification of individually useful therapy approaches. One possible consideration would be to repeat the questionnaire at certain intervals. In this way, it may already be possible to capture the dynamics of the complaints or the success/failure of treatment strategies and make appropriate adjustments. All in all, it can be said that all PROMs have their advantages and disadvantages and it is therefore difficult to speak of “the one” PROM.

Therefore, it is an urgent desire that the knowledge and understanding of CIPN is increased among physicians and in the society but also in insurance companies. In addition, there is a lack of prevention, effective treatments, or compensation strategies for patients to increase their ability to work or participate in leisure activities. There is also a need for bureaucratic help, for example, when applying for and approving rehabilitation measures or the recognition of being severely disabled.

In addition, effective measures to alleviate the symptoms are required. Many patients use a variety of therapeutic methods to achieve the best possible relief from their symptoms, mostly with little success.

In our survey, the individually implemented therapy measures were used most frequently and, from the patient’s point of view, most effectively to treat the complaints. The proven efficacy of duloxetine [16] and other antidepressants was slightly surpassed by pregabalin in our study, even though its efficacy could not be proven with certainty in other studies [16–18]. Although almost half of our patients attested painkillers a fairly good efficacy, the efficacy in comparative studies—among other things due to a decongestant effect with increased tissue pressure—is considered to be low [16]. Medication-related side effects must be considered; they can exacerbate typical complications of CIPN, such as an increased tendency to fall [16]. The efficacy of vitamin B12—the most frequently used drug in our study—is controversial from the patient’s point of view. It is predominantly considered to be ineffective or only slightly effective. Comparative studies could not confirm a statistically significant (preventive) efficacy by vitamin B12 [17, 19], as with the application of alpha-lipoic acid [17, 20, 21]. In our study, the strongest positive effect was achieved especially by individually performed physical mobilization and training, although these are not definitively recommended in other studies [22]. However, the discussed possibility that physical exercises can prevent and alleviate CIPN [10–12, 20, 21, 23–25] is supported by our study. Independently performed physical (strengthening) exercises also seem to have a positive effect on symptoms [23, 26, 27]. Therefore, their application should be intensified, promoted, and their application quality improved, for example, through patient training. They can thus act as an application tool that frees patients from their paralyzing feelings of powerlessness regarding CIPN and its symptoms and reduces their psychological burden. Patients could thus actively contribute to the improvement of their symptoms and quality of life and reduce their symptom burden [11, 18, 28].

In addition to an effective drug that has few side effects, the desire for long-term physiotherapeutic and ergotherapeutic treatment was often expressed. To achieve this, more studies to clearly define which methods or substances may help are necessary. Especially because many complementary or alternative therapy concepts [11] (e.g., change in diet [20, 29], (sound) massages [20], acupuncture [18, 20, 25, 29, 30], stimulation training) [21] are offered. Patients expressed a desire for more research on CIPN and prevention strategies in the future. The aim - when drug therapy is rejected - is  comprehensive treatment which is as effective as possible, especially since many patients are not convinced of the effectiveness of drugs in their situation. This also includes to consider the psychological stress [31] caused by the symptoms and the underlying malignant disease. Treatment of these psychological complaints should be offered or integrated into the multimodal therapy concept.

Due to the high level of suffering, many patients are willing to invest time and money in therapeutic methods on their own to alleviate symptoms. For this purpose, more information or guidelines on treatment approaches that can be carried out individually and independently would be desirable/useful.

Although early reporting of neurotoxic symptoms and early initiation of (electrophysical) diagnostics and therapy—e.g., adjustment of chemotherapy dose—are recommended when symptoms occur [32, 33], our study shows that it often still takes too long for a medical response and adjustment of therapy to occur. Here, further sensitization and education of both patients and medical staff, regarding recognition and therapy initiation, are needed. The use of standardized questionnaires and guidelines would be desirable [34] and one possible approach in order to react adequately to even the slightest symptoms and to avoid their worsening.

There is also much potential for improvement in medical care. Many patients feel left alone with their symptoms and often do not know which specialist is in charge for their treatment or would be best suited. They have the impression that their reporting of the complaints is not acknowledged, that they are trivialized or that the doctors are simply overwhelmed. Patients describe doctors’ contact as “the oncologist is not interested”; “no one feels responsible”; or “doctors do not respond.” In extreme cases, patients describe the impression that the treating specialists have no knowledge of the CIPN. As a result, patients report that they had to take care of applying for treatment and support on their own, with a great deal of effort. In order to ensure optimal treatment and care, improved interdisciplinary cooperation between the various medical specialties would be desirable.

## Limitations

There are two major limitations of our study. First of all, we addressed the participants via self-help organizations which may lead to a selection of active and engaged patients. Moreover, patients who feel much burden and frustration might be inclined more often to participate in such a survey. Second, the complaints were only assessed in the questionnaire on the basis of the self-assessment, not by objective examinations/clinical parameters.

Likewise, the various therapeutic approaches and their effectiveness were only determined by the subjective impression of the patient, not by objective clinical parameters. Their effect can therefore not be objectified, also due to the different (low) frequency of use. By focusing on CIPN as the cause of disease, it is possible that individual symptoms dependent on disease genesis and other differences were not considered. Therefore, it is possible that certain disease symptoms (due to their varying prevalence) were over- or underrepresented in our study. In addition, as a cross-sectional study, our study only allows a snapshot of symptoms, treatment response, etc. Therefore, it is not possible to assess whether a dynamic of the disease takes place. Further/specific investigations and studies would be necessary for learn more on the topic.

## Conclusion

Chemotherapy-induced polyneuropathy is a common side effect that usually affects patients in the long term. The clinical appearance is mostly similar in its basic features but is often also characterized by individual peculiarities. Everyday life is often negatively influenced or determined in a variety of ways, with many patients describing a situation-/time-of-day-dependent worsening of their symptoms. A generally applicable therapy that is effective for most patients is still lacking. Its importance has been repeatedly emphasized by the patients. A combination of medical, medicinal, and individual therapy methods is mainly used to treat symptoms, although the desired degree of relief is usually not achieved. This requires further optimization and research. The response to different treatments also varies from patient to patient. The attending physicians should consider this by their treatment, handling, and communication with the patients. For optimal treatment of the complaints, different therapies should therefore be tested participatory and critically evaluated in joint doctor-patient discussions. This requires comprehensive (improved) information about this side effect and most important strategies for prevention as sensomotoric training, even before the start of chemotherapy. In this way, patients should be “picked up” at their level of knowledge to avoid misunderstandings and increase patients’ satisfaction and quality of life.

## Supplementary Information

Below is the link to the electronic supplementary material.

Supplementary file1 (PDF 483 KB)

## Data Availability

The primary data from the survey are available from the first author on reasonable request.
