# Supplementary material for: Chemotherapy-induced polyneuropathy in cancer care—the patient perspective
Source: Support Care Cancer. 2023 Mar 27;31(4):235. doi: 10.1007/s00520-023-07688-5 (PMC10042917; doi:10.1007/s00520-023-07688-5)
Supplement: Supplementary file 1 — Supplementary file1 (PDF 483 KB) [file 520_2023_7688_MOESM1_ESM.pdf]

Dear Ms/Mr ....

Many patients suffer from a so-called polyneuropathy, i.e. a disturbance of the sense of touch, after treatment with anti-cancer drugs. This can have an influence on the everyday life of the affected person. We would like to learn more about this in our scientific study, because little is known about the extent and limitations of polyneuropathy.

Therefore, we would like to invite you to participate in a survey with an anonymous questionnaire.

Our questionnaire collects anonymized data. Answering the questions is voluntary and does not allow any conclusions to be drawn about your person. Participation also has no influence on your treatment.
However, by participating, you can help us to learn more about the significance of polyneuropathy and to develop methods of helping those affected.

In this survey, anonymized data is collected, stored and evaluated. The use of the data is in accordance with legal requirements and assumes your consent.

1. I agree that anonymous data about me will be collected in the course of this survey and stored in paper form as well as on electronic data carriers of password-protected computers for scientific evaluation.

2. Furthermore, I agree that authorized and competent monitoring authorities bound to secrecy may inspect the anonymized data as far as this is necessary for the verification of the proper conduct of the study.

3. I have been informed that my participation in the survey is voluntary and that I give my consent. The consent to the collection and processing of my anonymous data is irrevocable. Due to the anonymous collection of the data, the stored data can no longer be deleted retroactively

4. I agree that my data will be stored for at least ten years after completion of the survey. After this period, my data will be deleted unless there are legal or statutory retention periods to the contrary.

By completing and returning the questionnaire, you agree to participate in the survey.

With kind regards

Your Outpatient Clinic for Naturopathy and Integrative Oncology Jena
Headed by Frau Prof. Dr. med. Jutta Hübner

1. **Please tick your gender.**
   - ☐ Male
   - ☐ Female
   - ☐ Diverse

2. **Please indicate your age (in years)**

   _____ Years

3. **Approximately how long have the symptoms of polyneuropathy been present?**

Please specify the duration of years and (optionally) months.

____ Years ____ Months

4. **Was the polyneuropathy caused by tumor drugs (chemotherapeutic agents)?**
   - o Yes
   - o No, due to other medications/diseases
     - ☐ Diabetes
     - ☐ TNF-Inhibitors
     - ☐ Taking antidepressants
     - ☐ Other cause, namely ______________________________
     - ☐ Cause unkonwn

5. **What type of cancer was chemotherapy used to treat?**

   - ☐ Breas cancer
   - ☐ Ovarian cancer
   - ☐ Prostate cancer
   - ☐ Stomach cancer
   - ☐ Colorectal cancer
   - ☐ Pancreatic cancer
   - ☐ Lung cancer
   - ☐ Leukemia/Lymphoma
   - ☐ Other, namely

6. **Which initial changes or complaints did you notice in connection with the polyneuropathy disease?**
   Please describe them briefly in keywords

   ____

   ____

   ____

7. **Approximately how long did it take, from the first symptoms to the diagnosis of polyneuropathy?**
   Please indicate the number of months elapsed until then

From the first symptoms to the diagnosis, it took about _____ months.

8. **By which (specialist) doctor was the diagnosis of polyneuropathy made?**
   Please tick the (specialist) doctor who is applicable
   - ☐ Oncologist
   - ☐ Neurologist
   - ☐ Family docotor
   - ☐ Diabetologist
   - ☐ Other, namely ____

9. **Please mark in the left image (white background), where everywhere you experience insensations/sensitivity disturbances in the context of the polyneuropathy.**
   Afterwards, please mark in the right image (gray background) where you also/instead feel pain (due to the polyneuropathy).

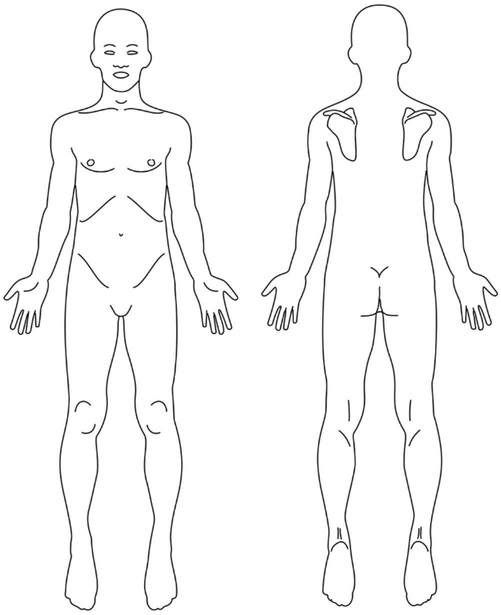

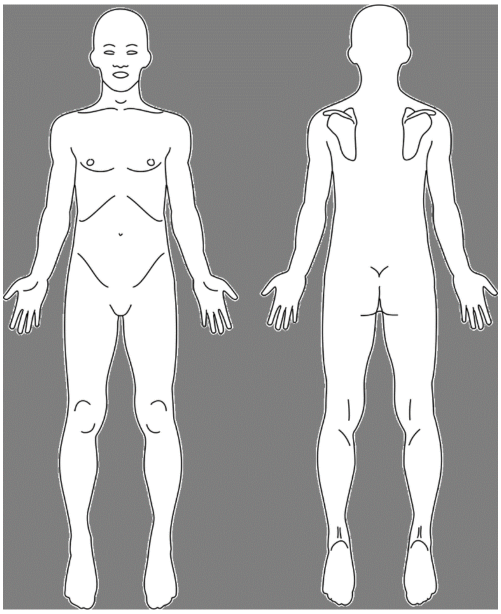

10. **Please tick (if any) the severity of pain experienced as part of the polyneuropathy condition.**
    If you do not have pain, please leave this question blank.
    - ☐ Weak/barely present
    - ☐ Mild
    - ☐ Moderately severe
    - ☐ Strong
    - ☐ Very strong
    - ☐ Unbearable

11. **Please tick which of the symptoms listed, you would use to describe your symptoms.**

Multiple selection is possible

Tingling sensation	
Cramping	
Burning	
(painful) stinging	
Numbness	
Feeling of pressure	
Extremities feel heavy	
Decreased sensibility	
Without looking Position of limbs unclear	
Short „freezing“of the affected limb	
Walking feels like being on “hot coals”	
Extremity feels (ice)-cold	
Other description, namely:	

12. **Please mark with a cross which complaints are associated with polyneuropathy for you and how strongly they influence your everyday life.**

Multiple selection is possible

	Low influence	Middle	Strong
Wakefulness			
Sleeping through disorders			
Pain with light (normally) non-painful touch			
Unintentional dropping of objects			
Fine motor skills disorders (grasping small things, cooking, needlework, etc.)			
Open/close buttons (on clothing)			
Increased hand injuries (e.g. when cutting nails)			
When shaving			
Lack of strength/reduced endurance			
Increased stumbling/falling or general balance problems (e.g. when climbing stairs)			
Unnoticed loss of worn shoes			
Increased number of foot injuries			
Fear of overexerting oneself and thereby aggravating the symptoms			
For hobbies, namely			
At work, namely			
Other complaints, namely			

13. **To what extent do you feel that the symptoms (of the polyneuropathy disease) affect/restrict you overall in your daily life?**

Please tick

- ☐ Not at all
- ☐ Now and then
- ☐ Frequent
- ☐ Always

14. Are the symptoms (of polyneuropathic disease) always the same?

- ☐ Yes
- ☐ No

15. Please tick in which situations and by how much the complaints become stronger/worse.

Multiple selection is possible

	Low	Moderate	Much
In the morning			
In the evening			
After getting up			
In bed/while relaxing			
During heat			
During cold			
Due to strong / much movement			
When touched/slightly bumped			
Due to stress/stressful situations			
In other situations, namely			

16. Please tick which medical therapies are used to treat your polyneuropathy and how helpful you think they are in treating your symptoms.

Multiple selection is possible

	Not helpful	Little	Some	Very
Physiotherapy				
Gymnastics				
Electricity therapy				
Lymphatic drainage				
Rehab				
Cure				
Psychological support				
Other treatment, namely: ___________				

17. **Please complete the medication names you are currently using to treat your polyneuropathy.**

Then please tick in each case when you take the medication and how effective you think it is at doing so.

	Taking as needed	Daily intake	Not helping	Hardly	Some	Strong
Pain killers (z.B. Ibuprofen, Paracetamol)						
Pregabalin						
Antidepressants/psychotropic drugs, namely:						
Alpha Lipoic Acid						
Vitamin B12						
Other drug, namely:						
Not remembering the exact name, sounded like:						

18. **Please tick which of the measures listed below you have already tried yourself to improve your symptoms and how helpful you consider them to be.**

	Not helping	Hardly	Some	Strong
Distraction (hobbies, work, meeting with family/friends, watching movies etc.)				
Talking with family/other affected patients about the complaints				
Exercise/sport				
Strengthening exercises				
Mobility/balance exercises				
Mental training/relaxation exercises				
Heat				
Cold				
Elevating of the feet				
Eye control during movement (avoiding falls or the dropping of objects)				
Other, namely:				

19. Do you think that more support is needed for polyneuropathy, its treatment and the resulting consequences?
Please briefly mention the relevant areas (if necessary)

- ☐ No
- ☐ Yes, at ____

20. Is there anything else you would like to see more support on, that needs to change or improve?
You are also welcome to write what you would like to see in terms of polyneuropathy (treatment) and/or what you would like to get rid of.

____

____

____

____

____

____

____

If you still have some time, we would be pleased if you voluntarily answer a few more questions regarding your polyneuropathy disease. These revolve primarily around the doctor-patient relationship and your satisfaction with it.
If you have time and interest in these additional questions, you will find them on the next two pages.
If you have no more time, we would like to thank you very much for your assistance!

21. Was polyneuropathy mentioned as a possible side effect in the explanatory talk before chemotherapy? Were the symptoms explained to you in a comprehensible and understandable way?

- ☐ Yes
- ☐ No
- ☐ Was mentioned
- ☐ I do not know anymore
- ☐ Different, namely ______________________________

22. Please check off the extent to which you were satisfied with your pre-chemotherapy education session.
Please give a brief explanation (in keywords).

- ☐ Completely satisfied because ______________________________
- ☐ Very satisfied because ______________________________
- ☐ Satisfied in most points because ______________________________
- ☐ Partially satisfied because ______________________________
- ☐ Satisfied in a few points because ______________________________
- ☐ Not satisfied at all because ______________________________

23. Please tick the (specialist) physicians who treat your polyneuropathy and how satisfied you are with their treatment.
Please tick only the doctors who are currently treating the complaints.

	Not at all	Rare	Often	Always
Oncologist				
Family doctor				
Neurologist				
Diabetologist				
Other (specialist) doctor, namely:				

24. Did you immediately address the complaints that occurred during chemotherapy with your attending (specialist) physician?
Please select the appropriate and complete them if necessary.

- ☐ Yes
- ☐ No, I addressed the complaints after _______ weeks, because:
  - o The complaints have become worse and worse
  - o Because the complaints did not get better/have stopped
  - o The inconvenience was no longer bearable
  - o Because family/friends have pushed for it
  - o ____
- ☐ No, I did not address the complaints because ____
- ☐ The complaints were addressed after _______ weeks by the attending physicians
- ☐ The complaints have come up quite differently, namely ____

25. How did your (specialist) doctors react to the description of your current complaints?
Please select the appropriate answer(s)

- ☐ Treated the complaints or searched for their cause
- ☐ Adjusted or stopped chemotherapy
- ☐ Help received only after specific request
- ☐ Ignored the complaints and continued the treatment unchanged
- ☐ Request for help was not heeded
- ☐ ____

Thank you for your participation!
